# Differential protein expression throughout the life cycle of *Trypanosoma congolense*, a major parasite of cattle in Africa

**DOI:** 10.1016/j.molbiopara.2011.02.009

**Published:** 2011-06

**Authors:** Brett A. Eyford, Tatsuya Sakurai, Derek Smith, Bianca Loveless, Christiane Hertz-Fowler, John E. Donelson, Noboru Inoue, Terry W. Pearson

**Affiliations:** a Department of Biochemistry and Microbiology, University of Victoria, Victoria, British Columbia V8W 3P6, Canada; b National Research Center for Protozoan Diseases, Obihiro University of Agriculture and Veterinary Medicine, Obihiro, Hokkaido 080-8555, Japan; c UVic-Genome BC Proteomics Centre, Vancouver Island Technology Park, Victoria, British Columbia V8Z 7X8, Canada; d Wellcome Trust Sanger Institute, Wellcome Trust Genome Campus, Hinxton CB10 1SA, UK; e Department of Biochemistry, Carver College of Medicine, University of Iowa, Iowa City, IA 52242, USA

**Keywords:** iTRAQ, isobaric tags for relative and absolute quantitation, PF, procyclic form, PCF, procyclic culture form, EMF, epimastigote form, MCF, metacyclic form, BSF, bloodstream form, VSG, variant surface glycoprotein, CESP, congolense epimastigote-specific protein, ORF, open reading frame, *Trypanosoma congolense*, Life cycle, Proteomics, Protein expression

## Abstract

*Trypanosoma congolense* is an important pathogen of livestock in Africa. To study protein expression throughout the *T. congolense* life cycle, we used culture-derived parasites of each of the three main insect stages and bloodstream stage parasites isolated from infected mice, to perform differential protein expression analysis. Three complete biological replicates of all four life cycle stages were produced from *T. congolense* IL3000, a cloned parasite that is amenable to culture of major life cycle stages *in vitro.* Cellular proteins from each life cycle stage were trypsin digested and the resulting peptides were labeled with isobaric tags for relative and absolute quantification (iTRAQ). The peptides were then analyzed by tandem mass spectrometry (MS/MS). This method was used to identify and relatively quantify proteins from the different life cycle stages in the same experiment. A search of the Wellcome Trust's Sanger Institute's semi-annotated *T. congolense* database was performed using the MS/MS fragmentation data to identify the corresponding source proteins. A total of 2088 unique protein sequences were identified, representing 23% of the ∼9000 proteins predicted for the *T. congolense* proteome. The 1291 most confidently identified proteins were prioritized for further study. Of these, 784 yielded annotated hits while 501 were described as “hypothetical proteins”. Six proteins showed no significant sequence similarity to any known proteins (from any species) and thus represent new, previously uncharacterized *T. congolense* proteins. Of particular interest among the remainder are several membrane molecules that showed drastic differential expression, including, not surprisingly, the well-studied variant surface glycoproteins (VSGs), invariant surface glycoproteins (ISGs) 65 and 75, congolense epimastigote specific protein (CESP), the surface protease GP63, an amino acid transporter, a pteridine transporter and a haptoglobin–hemoglobin receptor. Several of these surface disposed proteins are of functional interest as they are necessary for survival of the parasites.

## Graphical abstract

**Graphical abstract:**

**Figure:**
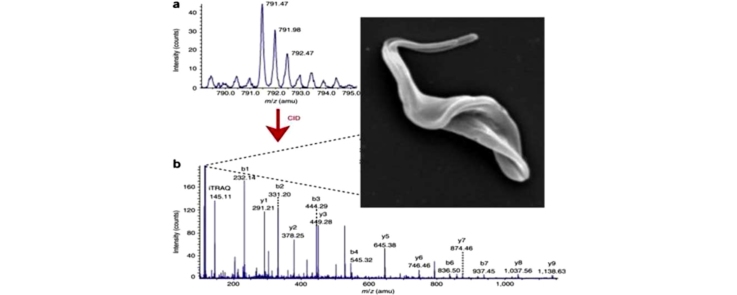

**Highlights:**

► All 4 major life cycle stages of *Trypanosoma congolense* were grown in the lab. ► Relative protein expression among the life cycle stages was studied by iTRAQ MS. ► Several known expression trends were observed and new patterns were identified. ► Special focus is given to lifecycle stage specific surface molecules. ► Six new proteins unique to *T. congolense* were discovered.

## 1 Introduction

Although there are vast natural resources in Africa, the human population of this continent is among the most impoverished in the world. The grasslands of Africa support thriving herds of large herbivores but raising domesticated livestock is hindered by zoonotic diseases. One of these diseases, animal African trypanosomiasis (AAT), known locally as n’gana, has a particularly devastating impact on African agriculture. The Food and Agriculture Organization of the United Nations has acknowledged that both AAT and human African trypanosomiasis are major impediments to the agricultural and economic development of Africa [1,2].

Trypanosomiasis in both humans and cattle is caused by kinetoplastid parasites of the genus *Trypanosoma*, which are spread by the bite of the infamous tsetse (*Glossina*). Tsetse flies inhabit 8.7 million km^2^ of sub-Saharan Africa known as the “tsetse belt”. This area represents approximately a third of Africa, or to put it into perspective, an area greater than that of the entire Australian continent. In the areas where tsetse are prevalent, agricultural output is suboptimal because of the risk of AAT. Every year approximately 40 million cattle are threatened and 3 million are killed by trypanosomiasis. The economic loss resulting directly from animal death is in the range of US$ 1.0–1.2 billion annually. When secondary losses such as reduced manure and draft power and thus decreased crop yields are included, the total gross domestic product lost can be as much as $4.5 billion per annum [2].

AAT is a collection of symptomatically similar diseases caused by a number of different trypanosome species. In cattle, the most widespread and virulent of these species is *Trypanosoma congolense*. In addition to cattle, *T. congolense* infects many other domesticated animals including sheep, pigs, goats, horses and camels and causes a chronic wasting (cachexia); characterized by anemia, weight loss and immunosuppression. The disease is fatal if untreated.

Pathogenic African trypanosomes, including *T. congolense*, have a complex developmental cycle that involves both mammalian hosts and tsetse vectors. In the mammalian host, bloodstream forms (BSF) are covered with a dense coat consisting of approximately 10^7^ copies per cell of VSG that is famously involved in antigenic variation, allowing the trypanosome population to avoid elimination by the host immune system [3]. BSF rely entirely on glycolysis and substrate level phosphorylation to meet their energy requirements and the single, large mitochondrion is almost entirely inactive during this stage of their life cycle.

When BSF trypanosomes are consumed by a tsetse during its blood meal, they enter the insect midgut and a subpopulation differentiates to procyclic forms (PF) that are adapted to life in the midgut [4]. Differentiation from BSF to PF is characterized by a switch to proline oxidation and oxidative phosphorylation for energy production. Procyclic trypanosomes can also be cultured in the laboratory *in vitro* as procyclic culture forms (PCF) [5]. PCF, like PF, use proline as their preferred energy source, although the typical media used to culture the parasites also contain glucose, which is absent or at very low levels in the tsetse vector. As a consequence, PCF still retain some level of glycolytic activity that is not present in their PF counterparts. Nevertheless, PCF are thought to be extremely similar to PF based on a variety of studies including comparison of their protein expression profiles [6]. Upon differentiation to procyclic forms, both *in vitro* and *in vivo* in the fly midgut, the VSG coat is replaced by a set of insect form specific surface molecules [7]. Once established in the fly, a small subset of the PF migrate from the midgut and begin to differentiate, becoming non-motile epimastigote forms (EMF) that adhere to the fly's salivary glands (*T. brucei*) or to the fly's proboscis and mouth parts (*T. congolense*). Finally, the dividing, adherent EMF differentiate into non-dividing, non-adherent, VSG-expressing and infectious metacyclic forms (MCF) and are injected into the host bloodstream when tsetse take a blood meal. The parasites multiply at the site of the fly bite for 5–9 days after which they invade the bloodstream and lymphatic system, becoming BSF and thus completing their life cycle.

Both *T. brucei* and *T. congolense* thus undergo cyclical transmission and interact with both tsetse vectors and mammalian hosts. These interactions influence parasite differentiation, survival, maturation and infectivity and are thus critical for parasite transmission and perpetuation of disease. Several molecules involved in these processes have been well studied, including metabolic enzymes and some surface molecules, especially VSGs in BSF. More recently, molecules of PF that are involved in tsetse–trypanosome interactions have received more attention [8]. One interesting aspect of cyclical transmission is the extreme constraints or bottlenecks placed on trypanosomes in the tsetse vectors and in transmission to mammals [8,9], although the molecular mechanisms involved are almost completely unknown.

*T. congolense* causes an economically and socially important disease. However, beyond some general information about enzymes involved in metabolism and the description of the few surface coat proteins, little is known about protein expression during the *T. congolense* life cycle. Transcriptome analysis has revealed much about differential gene expression in the four major life cycle stages of *T. congolense* [10]. However, protein expression in trypanosomes is strongly regulated post-transcriptionally [11]; thus, changes in mRNA abundance are not necessarily reflected at the protein level. Therefore, to form a better picture of changes that occur during *T. congolense* differentiation, we elected to study protein expression directly. We elected to use a fly-transmissible clone, *T. congolense* IL3000 [12,13]. All of the major life cycle stages of this parasite can be grown *in vitro* in sufficient quantities for biochemical analysis; thus it is ideally suited for proteomics and provides a unique opportunity to study differential protein expression throughout an infection cycle that is not currently possible for other trypanosome species.

**Fig. 1:**
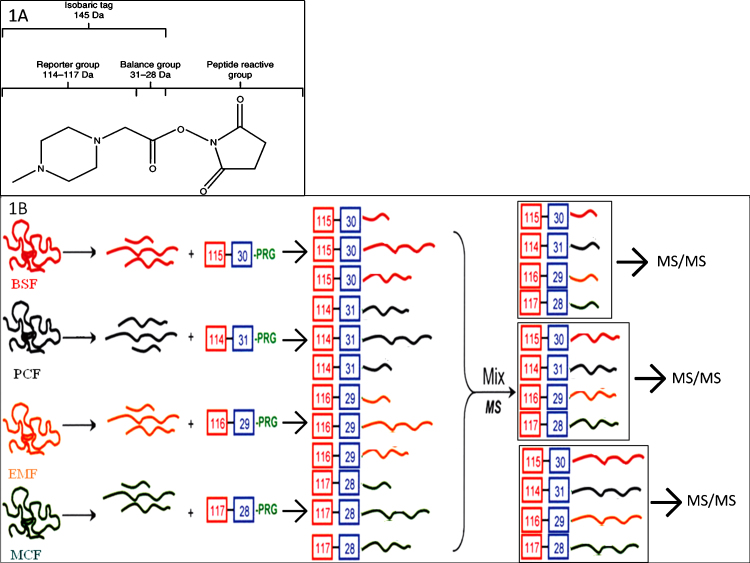
(A) The iTRAQ reagent is an isobaric tag consisting of a charged reporter group, a peptide-reactive group and a neutral balance portion. The reporter group ranges in mass from *m*/*z* 114 to 117, whereas the balance group ranges in mass from 28 to 31 Da, such that the combined mass remains constant (145 Da) for each of the four reagents. The figure was taken with permission from Ref. [40]. (B) A simplified schematic overview of the iTRAQ sample preparation and experiment. Proteins from each life cycle stage were digested to tryptic peptides and labeled with different isobaric tags in parallel prior to mixing and simultaneous MS analysis. The figure was modified with permission from Ref. [41].

To follow protein expression changes throughout the *T. congolense* life cycle, we used iTRAQ (AB SCIEX, Foster City, CA) in conjunction with tandem mass spectrometry (MS/MS). This method is used to identify and to determine relative quantification of proteins from different sources in the same experiment. The method is based on the covalent labeling of free amines of peptides, produced by tryptic digestion of protein samples, with isobaric tags that can be fragmented to release reporter ions of varying masses (Fig. 1A). Peptides with different mass tags can be subsequently identified by mass spectrometry. An overview of the method is shown in Fig. 1B.

**Fig. 2:**
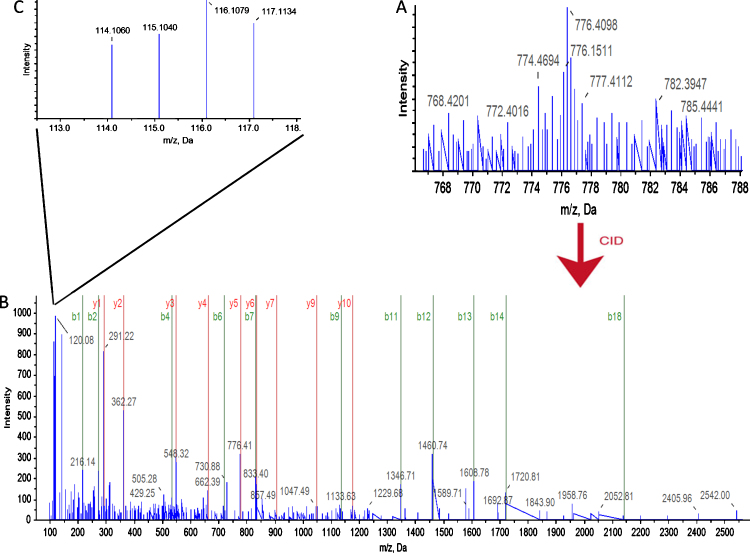
Example of a mass spectrum for a single iTRAQ labeled peptide. (A) Precursor ion scan (MS) of a peptide labeled with the iTRAQ reagent. The differentially labeled forms are indistinguishable and contribute to the same peak. (B) Product ion scan (MS/MS) of the fragmented iTRAQ-labeled peptide. The *b* and *y* ions are indicative of the peptide sequence. (C) Enlarged region of the product ion spectrum, with the iTRAQ reporter ions indicating the relative levels of peptide in the four original samples. The figure was compiled from spectra 16.1.1.3838.2 of replicate number one. The source protein is β-tubulin.

For our experiments four iTRAQ mass tags were used, one for each life cycle stage. The labeled peptide mixtures from each of the four life cycle stages were then pooled, fractionated by liquid chromatography and analyzed by MS/MS. A database search was performed using the MS/MS fragmentation data to identify the corresponding source proteins. The mass spectrometric fragmentation of the attached iTRAQ tags generates low molecular mass reporter ions (from 114 to 117 Da) that can be used to determine the relative amounts of the peptides and thus the relative abundance of the proteins from which the peptides originated (Fig. 2).

As a database, we used a semi-annotated open reading frame (ORF) library from *T. congolense* IL3000, one of the trypanosomes sequenced as part of the Sanger Institute's genome sequencing project: http://www.sanger.ac.uk/Projects/T_congolense/. As part of the iTRAQ differential protein expression analysis, we obtained a global list of expressed proteins. Since few proteins from *T. congolense* have been identified or characterized, this larger list of proteins was used to aid the annotation of the *T. congolense* genome by providing direct protein evidence for the existence of particular ORFs. In addition, we obtained differential protein expression data from all four *T. congolense* life cycle stages. We here report a protein expression analysis with a focus on surface molecules of trypanosomes, our primary interest.

## 2 Materials and methods

### 2.1 Trypanosomes and culture conditions

The four major life cycle stages of *T. congolense* clone IL3000 were prepared essentially as previously described [12] although since these life cycle stages are central to the work reported in this manuscript, we here describe their preparation in some detail. BSF stabilates of a cloned population of *T. congolense* IL3000 (Savannah group) [12,13], obtained from the International Livestock Research Institute (Nairobi, Kenya), were stored in liquid nitrogen at the National Research Center for Protozoan Diseases, Obihiro University of Agriculture and Veterinary Medicine, Obihiro, Hokkaido, Japan. To grow BSF, frozen stabilates were thawed and aliquots were inoculated intraperitoneally into five, 8 week old, female BALB/c mice. Infections were monitored microscopically using tail blood samples. At the first peak of parasitemia the mice were bled by cardiac puncture and trypanosomes were purified using DE-52 cellulose (Whatman, Middlesex, UK) anion exchange column chromatography [14]. All animal experiments were performed according to the standards for Care and Management of Experimental Animals at Obihiro University of Agriculture and Veterinary Medicine (No. 21-88).

Cultures of PCF, EMF and MCF were produced *in vitro* from the IL3000 BSF grown *in vivo* essentially by following the methods of Hirumi and Hirumi [15]. BSF were adjusted to 3 × 10^6^ cells/mL in Eagle's minimum essential medium containing 20% heat inactivated fetal bovine serum, 2 mM l-glutamine and 10 mM l-proline and 10 mL of this suspension were incubated in T-25 type culture flasks at 27 °C for approximately one week to allow differentiation to PCF. The PCF were maintained in log-phase of growth for several weeks to allow complete differentiation to PCF. For production of EMF, cultures of PCF were allowed to become slightly acid (overgrown) and after 1–2 months, adherent clusters of parasites began to appear on the surfaces of the flasks. Once these adherent cells were confluent, they were washed to remove non-adherent parasites before harvesting as EMF. As judged by light microscopy and the position of the kinetoplast, these parasites were clearly EMF although a few contaminating PCF were observed. After a few weeks of sustained incubation of flasks with confluent parasites, non-adherent, VSG-expressing MCF began to appear in the supernatant. To obtain the different life cycle stages prior to solubilization and iTRAQ labeling, different cultures were used. PCF were collected from suspension cultures of PCF growing in log-phase. EMF were collected from flasks containing confluent monolayers by first gently washing away loosely bound cells (PCF and emerging MCF) using 3 × 10 mL phosphate-buffered saline containing 1% glucose (PSG), followed by gently scraping the adherent cells from the culture flasks and resuspending them in PSG. MCF were purified from EMF culture supernatants by anion exchange chromatography as described above for BSF. MCF, which express surface VSG, pass through the column, while PCF and EMF cells are retained. Finally, the purified MCF were used to inoculate BALB/c mice (10^5^ parasites per animal) intraperitoneally and approximately seven days after infection, the infected mouse blood was collected by cardiac puncture and the BSF purified using DE-52 column chromatography. These BSF were used to continue the life cycle through two more complete cycles in the same manner as described above. A total of 12 cell isolates (4 life cycle stages, 3 replicates of each) were obtained over a year long period. At harvest, parasites from all four life cycle stages were concentrated by centrifugation at 1300 × *g* for 10 min at room temperature and washed three times with PSG before microscopic examination of cell morphology, counting and solubilization (see below).

### 2.2 Parasite collection and protein solubilization

Each washed cell preparation was suspended in PSG to 1010 μL. Ten μL of the cell suspension were used for counting the parasite numbers using a hemocytometer. The remaining parasite suspension was centrifuged (5000 × *g*, 5 min, 4 °C) and the supernatant removed. The pellets were re-suspended to a total volume of 1.0 mL in chilled lysis buffer (4.5 M urea, 0.2% SDS) and sonicated on ice for 1 min at a setting of 1.0 on a Model 100 Sonic Dismembranator (Fisher Scientific) fitted with a 5 mm diameter sonic probe. Lysates were frozen at −80 °C until all cell samples had been collected and were ready to process for iTRAQ analysis.

### 2.3 Protein quantitation, tryptic digestion and peptide labeling

The protein concentration of each sample was measured using a Bicinchoninic Acid kit (BCA1-1KT; Sigma–Aldrich, St. Louis, MO) using bovine serum albumin as a protein standard. One hundred μg of protein were precipitated by adding 9 volumes of ice cold acetone and incubating overnight at 4 °C. The precipitate was pelleted by centrifugation at 16,000 × *g* for 10 min and after removal of the acetone, the proteins were re-suspended in 30 μL 0.5 M triethyl ammonium bicarbonate containing 0.2% SDS. The proteins were then reduced, alkylated and digested with sequencing grade modified porcine trypsin (Cat No V511A; Promega, Madison, WI) as described in the iTRAQ^®^ Reagents Multiplex Kit (Cat. No. 4352135, AB SCIEX, Foster City, CA, USA). The resulting tryptic peptides were finally labeled with isobaric tags according to the instructions of the manufacturer (BSF peptides were labeled with iTRAQ tag 115; PCF peptides with tag 114, EMF peptides with tag 116 and MCF peptides with tag 117). The labeled peptides from each of the 4 life cycle stages were combined to form a single biological replicate. This was repeated for each set of the four life cycle stages to yield 3 peptide mixtures called replicates 1, 2 and 3, representing 3 complete biological replicates of the *T. congolense* life cycle.

### 2.4 Strong cation exchange (SCX) high performance liquid chromatography (HPLC)

Labeled peptides were subjected to SCX HPLC prior to MS/MS analysis. A Vision Workstation (AB SCIEX, Foster City, USA) was equipped with a polysulfoethyl A, 100 mm × 4.6 mm, 5 μm, 300 A SCX column (Poly LC, Columbia, MD). iTRAQ labeled peptide mixtures were brought up to 2 mL in buffer A (10 mM KPO_4_, pH 2.7/25% ACN) and injected onto the column. The column was allowed to equilibrate for 20 min in buffer A before a buffer gradient (0–35% buffer B; 10 mM KH_2_PO_4_, 25% ACN, 0.5 M KCl) was applied over 30 min at a flow rate of 0.5 mL/min. Fractions were collected at 1 min intervals after injection. The collected fractions were then reduced in volume to 125 μL in a Speed-Vac vacuum concentrator (Savant Instruments, Holbrook, NY) and transferred to autosampler vials (LC Packings, Amsterdam).

### 2.5 Liquid chromatography and tandem mass spectrometry (LC–MS/MS)

LC–MS/MS analysis was performed using an integrated Famos autosampler, Switchos II switching pump and UltiMate micro pump (LC Packings, Amsterdam) system coupled to a Hybrid Quadrupole-TOF LC/MS/MS Mass Spectrometer (QStar Pulsar i; AB SCIEX) equipped with a nano-electrospray ionization source (Proxeon, Odense, Denmark) and fitted with a 10 μm fused-silica emitter tip (New Objective, Woburn, MA). Chromatographic separation was achieved on a 75 μm × 15 cm C18 PepMap Nano LC column (3 μm, 100 Å, LC Packings, Amsterdam). A 300 μm × 5 mm C18 PepMap guard column (5 μm, 100 Å, LC Packings, Amsterdam) was in place before switching in line with the analytical column and the MS. The mobile phase (solvent A) consisted of water/acetonitrile (98:2, v/v) with 0.05% formic acid for sample injection and equilibration on the guard column at a flow rate of 100 μL/min. A linear gradient was created upon switching the trapping column inline by mixing with solvent B which consisted of acetonitrile/water (98:2, v/v) with 0.05% formic acid and the flow rate was reduced to 200 nL/min for high resolution chromatography and introduction into the mass spectrometer.

Twenty seven μL of each Speed-Vac concentrated sample was injected in 95% solvent A and allowed to equilibrate on the trapping column for 10 min to wash away any contaminants. Upon switching inline with the MS, a linear gradient from 95% to 40% solvent A was developed for 40 min and in the following 5 min the composition of mobile phase was decreased to 20% A before increasing to 95% A for a 15 min equilibration before the next sample injection. MS data were acquired automatically using Analyst QS 1.0 software Service Pack 8 (ABI MDS SCIEX, Concord, Canada). An information dependent acquisition method was used, consisting of a 1 second TOF-MS survey scan of mass range 400–1200 amu and two 2.5 second product ion scans of mass range 100–1500 amu. The two most intense peaks over 20 counts, with charge state 2–5 were selected for fragmentation and a 6 amu window was used to prevent the peaks from the same isotopic cluster from being fragmented again. Once an ion was selected for MS/MS fragmentation it was put on an exclude list for 180 s. Curtain gas was set at 23, nitrogen was used as the collision gas and the ionization tip was set to 2700 V. If the observed *A*_215_ was greater than 0.1 for any fraction collected during the SCX, a 2.5 h gradient (95–50% solvent A) was used to compensate for the higher peptide concentration in that fraction.

### 2.6 Data processing and analysis

Using ProteinPilot V2.0.1 (AB SCIEX, Foster City, CA, USA), a semi-annotated *T. congolense* translated genome database of 13,485 ORFs (Sanger Institute) was trypsin digested *in silico* and the MS/MS fragmentation patterns were predicted for all resulting peptides. The tryptic peptides (and parent proteins) were identified by comparing the predicted MS/MS peptide fragmentation patterns with those observed during the iTRAQ experiments. Proteins were included in the results file if they had a total score representing ≥95% confidence of a correct identification. Further confidence about the accuracy of protein identification was gained when a protein was identified in more than one biological replicate. A webinar at http://appliedbiosystems.cnpg.com/lsca/webinar/proteinpilot/20060516/ provides more information about the ProteinPilot software. Technical variation among iTRAQ replicates has been well studied and is known to be minimal when properly performed [16]. The sequences for all proteins described in this manuscript can be found by their accession number at tritrypdb.org.

The ProteinPilot software then calculated the ratios for the iTRAQ reporter ion fragment masses for each peptide. The relative abundance of each reporter ion was used to calculate the relative abundance of the peptide from each life cycle stage. The cumulative strength of the reporter ions from all the peptides assigned to a given protein represents the relative abundance of that protein in one life cycle stage when compared to another.

An excel spreadsheet with the condensed iTRAQ results as well as the raw ProteinPilot files are included in the supplementary material. All expression changes shown in the tables and supplemental Excel sheets are normalized against the previous life cycle stage. For example, a number greater than 1 in a column labeled BSF → PCF means that the given protein is expressed to a higher level in PCF compared to BSF.

### 2.7 Immunoblotting

Immunoblotting was performed for relative quantitation of selected proteins in each life cycle stage of trypanosomes in order to support the iTRAQ data. Parasite proteins in lysates (grown as described above and representing a 4th biological replicate) were separated by SDS–PAGE gel electrophoresis followed by transfer to polyvinylidene difluoride (PVDF) transfer membrane (Immobilon™-P, Millipore, Billerica, USA) as previously described [17]. The primary antibodies used were: a 1:2000 dilution of ascites fluids containing the murine mAb 1-G10 (specific for trypanosome β-tubulin; TWP lab, unpublished), a 1:2000 dilution of ascites fluid containing the murine mAb CLP007A (Cedarlane, Burlington ON) specific for major lysosomal membrane protein p67 [18], 1:1 dilutions of hybridoma supernatants containing mouse anti-trypanosome glycerol-3-phosphate dehydrogenase (GPD) [19], anti-glutamic acid/alanine rich protein (GARP) [17], anti-CESP (BL and TWP, unpublished), or anti-flagellar calcium binding protein (BE and TWP, unpublished), originally described as a *T. congolense* specific marker of unknown identity [20]. The secondary (detecting) antibody used was a 1/20,000 dilution of horseradish peroxidase conjugated goat anti-mouse IgG/IgM (H+L) (Caltag Laboratories, South San Francisco, CA). The substrate used was SuperSignal West Dura (Thermo Scientific, Rockford, IL). Kodak Biomax MR film (Eastman Kodak Company, Rochester, NY) was used to detect chemiluminescence. After development of the autoluminograms, proteins were stained on the PVDF membrane with 0.2% (w/v) nigrosin in PBS to ensure equivalent protein loading.

## 3 Results and discussion

### 3.1 Identification of proteins by iTRAQ

**Fig. 3:**
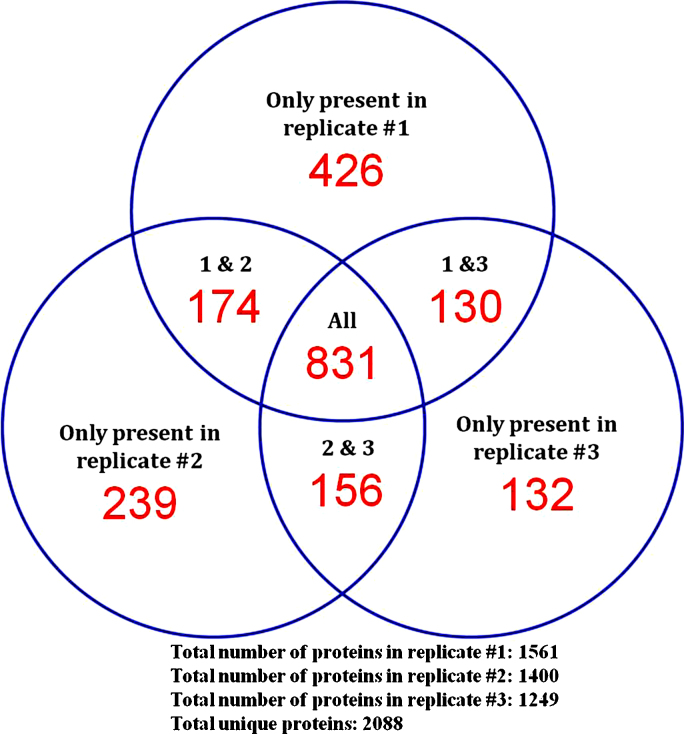
Venn diagram showing the distribution of unique protein hits among the 3 biological replicates of the iTRAQ experiments.

In the present study, all four *T. congolense* life cycle stages were produced on three separate occasions over a year long period, representing three complete biological replicates. With each replicate, the parasite proteins were solubilized and quantitated and 100 micrograms from each life cycle stage were reduced, alkylated and digested with trypsin to produce peptides for iTRAQ labeling. After peptide separation by strong cation exchange and reverse phase liquid chromatography and MS/MS analysis, a total of 138,787 spectra were collected, resulting in determination of 61,410 peptide sequences. For each peptide sequence, a score was calculated that represents the confidence with which that peptide had been identified correctly. These peptides were assigned to parent proteins. The confidence score for each protein being the cumulative score/confidence of the peptides assigned to it. Only those proteins with a sum peptide score representing ≥95% confidence are included in the iTRAQ analysis results. In this way, 1561, 1400, and 1249 proteins were identified in replicates one, two and three, respectively. A total of 831 proteins were identified in all 3 replicates, whereas 460 were present in 2 of the 3 replicates and 797 were only observed in a single replicate. A breakdown of protein identification is shown in the Venn diagram (Fig. 3). A total of 2088 unique protein sequences were identified, representing 23% of the ∼9000 proteins predicted for the *T. congolense* proteome.

For analysis of the MS/MS data by ProteinPilot software, the DNA ORF sequences in the *T. congolense* genome database were translated into protein sequences. The genome sequence had undergone preliminary annotation; thus, some of the proteins identified by the iTRAQ experiments were already annotated. However, most ORFs were designated as “undefined products”. To further the annotation of the *T. congolense* proteome and genome, the protein sequences identified by iTRAQ were queried by BLAST against the global non-redundant database. Not surprisingly, many proteins drew strong hits with known, characterized proteins of kinetoplastids including *T. congolense*, *T. brucei*, *T. cruzi*, and *Leishmania*. However, many proteins, although drawing hits from other kinetoplastids, were only labeled as “hypothetical proteins” resulting from genome sequencing efforts. Such proteins have been directly observed by our iTRAQ-MS/MS experiments meaning that the designation “hypothetical” no longer applies.

The convention in the literature has been to identify a given protein in two separate iTRAQ experiments performed on biological replicates (each at ≥95% confidence) before protein identification can be considered significant. Therefore, to ensure that our results were reliable, only proteins that were observed in 2 or 3 replicates were considered for in depth analysis at this time. A total of 1291 proteins, representing approximately 14% of the *T. congolense* proteome, met these criteria and are the focus of the sections below.

### 3.2 Validation of iTRAQ expression data

**Fig. 4:**
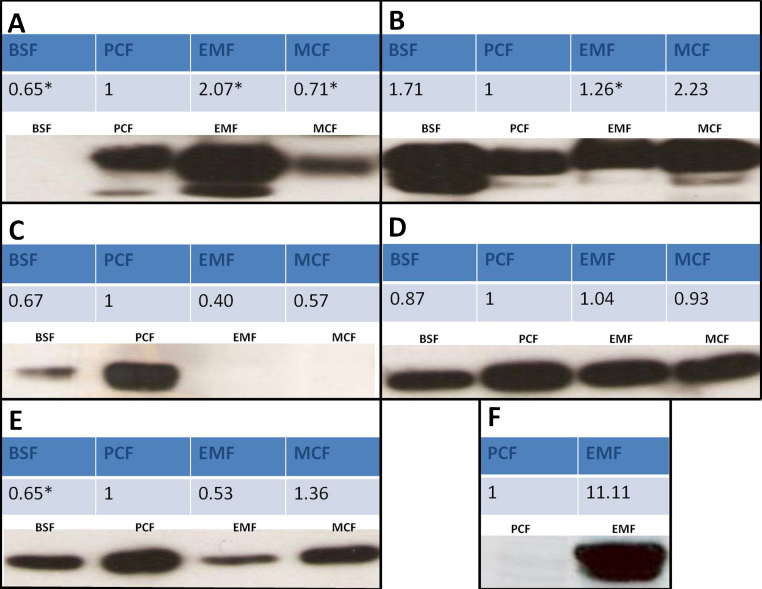
Immunoblots on *T. congolense* cell lysates using various monoclonal antibodies. The inserted iTRAQ expression data shown represent the average of all reliable replicates (in some cases data with a *p*-value > 0.05 were included, averages that have some uncertain data included are marked with an asterisk) and normalized so that the expression level in PCF always equals 1. (A) Glutamic acid/alanine rich protein. (B) Major lysosomal membrane protein, p67. (C) Glycerol-3 phosphate dehydrogenase. (D) β tubulin. (E) Flagellar calcium binding protein. (F) Congolense epimastigote specific protein. The multiple or smeared bands observed in panels A, B and F are possibly due to glycosylation or other post-translational modifications of the proteins.

Before the iTRAQ expression data were analyzed in depth we first wanted to determine whether the expression trends observed by iTRAQ could be corroborated through a different experimental method. Immunoblotting was chosen to confirm some of the iTRAQ expression results because our lab at the University of Victoria has several well characterized monoclonal antibodies that specifically recognize different proteins identified by our mass spectrometric analysis. The results are shown in Fig. 4. The immunoblot results paralleled the iTRAQ expression data for all five proteins examined.

The major *T. congolense* surface molecule GARP [17,21] was shown by iTRAQ analysis to be most strongly up regulated in EMF followed by PCF and this was confirmed by immunoblotting (Fig. 4A). Transcriptome analysis [10] has shown that GARP exhibits high expression levels in EMF with lower expression in PCF and this was clearly observed here. It is interesting that the MCF continued to express low levels of GARP, perhaps indicating that although these parasites already expressed VSG they were not fully differentiated from EMF.

The major lysosomal membrane protein, p67, [18] was more highly expressed in the mammal-infective BSF and MCF parasites according to both iTRAQ and immunoblot methods (Fig. 4B). The monoclonal antibody used in this immunoblot recognizes a carbohydrate specific to p67. In *T. brucei* the core polypeptide is expressed in both BSF and PCF. However the antibody binds only the BSF protein due to changes in post-translational processing upon differentiation to PCF. The fact that this antibody is able to bind to an antigen in *T. congolense* PCF would seem to indicate that this example of life cycle stage differential glycosylation is not evident in this species of trypanosome.

By iTRAQ analysis, GPD was expressed in all four life cycle stages but was expressed at a level 1.5–2.5 fold higher in PCF than in any other stage (Fig. 4C). Only PCF and to a lesser extent, BSF, expressed significant amounts of GPD as detected by immunoblotting. Although the trends observed by iTRAQ and immunoblot are consistent with each other, these results are somewhat unexpected. GPD is an enzyme known to help maintain glycosomal redox balance during glycolysis in BSF. Although GPD has been described as having high activity in BSF, previous reports have also indicated that it is present in all life cycle stages at roughly equal levels [19].

Trypanosome β-tubulin was expressed at approximately equal levels in all four major life cycle stages as determined by both iTRAQ and immunoblotting (Fig. 4D). Although the different trypanosome life cycle stages show different sizes and shapes, perhaps with different amounts per cell of β-tubulin, this structural protein remains relatively constant when based on total protein as was done for the current iTRAQ analysis.

To our knowledge there is no previous literature describing the *T. congolense* flagellar calcium binding protein (aka calflagin or FCaBP) which is depicted in Fig. 4E. Homologues of this EF hand, acyl switch protein have been described in other trypanosomatids such as *T. brucei* and *T. cruzi* [22,23]. Calflagin is known to act as a calcium sensor with effects on flagellar function and assembly. Calflagin is notably immunogenic and has been proposed as a target for the serodiagnosis of Chagas’ disease caused by *T. cruzi* infection [24]. It has also been found that depleting calflagin by RNA interference can reduce parasitemia and prolong survival in mice infected with *T. b. brucei*. The *T. congolense* calflagin may be of interest as a drug target as well [25].

The surface membrane molecule, CESP [26] was strikingly up regulated in EMF as determined by both iTRAQ (11.1 fold) and by immunoblotting where it was not detected at all in the immediately preceding PCF stage (Fig. 4F) nor in BSF and MCF stages (not shown). CESP was also measured in flow cytometry and immunofluorescence experiments in parallel with immunoblotting on all four life cycle stages and was found to be expressed only in EMF parasites (BL, unpublished).

Another (albeit indirect) means for validating the iTRAQ results can be achieved by comparison of the iTRAQ expression data with previously well established information about the VSGs which are considered a hallmark of MCF and BSF parasites that are infective for mammals. VSGs constitute the major surface coat on these life cycle stages and are responsible for the phenomenon of antigenic variation whereby the trypanosomes avoid immune elimination from host mammals. MCF parasites of both *T. congolense* [27] and *T. brucei* [28] express one of about 12–15 different VSGs (mVSGs), whereas BSF parasites can sequentially express more than one hundred different VSGs, enabling them to evade the host immune response [29]. The sequence of the IL3000 VSG and several mVSGs of the *T. congolense* IL3000 clone have been deduced previously from expressed sequence tag (EST or cDNA) clones in EST libraries of the four life cycle stages [10]. Our iTRAQ data showed that eleven different VSGs were expressed in at least some biological replicates of the different trypanosome life cycle stages (Table 1, ordered by accession number). Four were confidently determined to be expressed at higher levels in MCF than in BSF, two of which correspond to mVSGs 3 and 6 previously identified from ESTs in a MCF EST library [10]. Five more VSGs also showed increased expression in MCF but these data came from only one replicate and may be less reliable. One of these VSGs, (TcIL3000.0.12850 in Table 1) is the same as the previously identified mVSG10 [10]. Two VSGs were confidently expressed at higher levels in BSF, one of which is the expected IL3000 VSG from which *T. congolense* IL3000 receives its name (Table 1). The other BSF VSG (TcIL3000.0.41540 in Table 1) has not been detected previously and its detection is most easily explained by the possibility that some of the IL3000 BSF parasites had switched to the expression of another bloodstream VSG. The data are believable since this VSG was detected in all 3 independent biological replicates. In addition to the patterns described above, 5 VSGs showed moderately increased expression (1.45–1.76 fold) in EMF compared to PCF. This observation may indicate that the EMF populations used for our iTRAQ experiments were not homogenous or alternatively, that some VSG expression had started to occur prior to surface expression on MCF. Since trypanosome differentiation through the different life cycle stages occurs as a continuum, the latter is a distinct possibility.

### 3.3 Discovery of unidentified *T. congolense* proteins

Six different proteins identified by our iTRAQ analysis showed no significant sequence similarity with proteins in the non-redundant database (Table 2). One of these, TcIL3000.0.22490 showed no hit at all. Unfortunately there were no reliable expression data associated with this protein. A second unidentified protein (TcIL3000.0.28430), showed a 17.9 fold increase when MCF differentiated to BSF and then a 14 fold drop when the BSF transformed to PCF. This protein deserves examination as it is so highly expressed in BSF. A third protein (TcIL3000.0.38630) increased 8.5 fold during differentiation from EMF to MCF, then decreased 1.6 fold in BSF and 6.7 fold in PCF; thus, it appears to be mainly expressed in metacyclic forms with lowest expression in PCF. In contrast, a fourth protein (TcIL3000.0.51090) was most highly expressed in PCF. It dropped 5.3 fold during differentiation from PCF to EMF, dropped 6 fold further in MCF, then rose 5.8 fold in BSF and 5.1 fold more in PCF. No expression data were obtained for the fifth protein (TcIL3000.2.410). Finally, the sixth protein (TcIL3000.7.3440) was more highly expressed in EMF (5.44-fold up regulated from PCF) than any other life cycle stage. All of these molecules offer possibilities for study as none have been previously identified and their functional roles are undefined.

### 3.4 Surface membrane proteins

It is not surprising that several of the *T. congolense* surface membrane proteins known to exhibit stage-specific expression also showed stage-specific expression revealed by iTRAQ analysis. In MCF and BSF trypanosomes that are infective for mammals, a total of 11 different VSGs were detected (see discussion above under Section 3.2 and Table 1).

In addition to VSG expression, four proteins with sequence similarity to ISG65 and ISG75 [30] of *T. brucei* were found (the two with good expression data are shown in Table 3). Analysis of the *T. brucei* molecules revealed that both amino acid sequences predict polypeptides with N-terminal signal sequences and large extracellular domains [30]. These glycoproteins have no sequence similarity to known proteins in non-trypanosomatids and after VSGs are likely the most abundant surface proteins in mammal infective forms. The *T. brucei* ISGs have been shown to be spread over the entire cell surface, although they are present at 50–70,000 copies per cell, approximately 0.5% of the VSG copy number [30]. These glycoproteins have been proposed to be dispersed between VSG molecules on the trypanosome surface [31]. The genes encoding ISG75 in at least one strain of *T. brucei* exist as seven copies at two loci, suggesting that different isoforms may be important for the survival of the parasite. In our iTRAQ study, proteins with amino acid sequence similarity to the ISGs were most highly up regulated in MCF. Perhaps ISG isoforms are differentially regulated in this life cycle stage. *T. brucei* MCF have not been previously examined for ISG expression and neither have *T. congolense* MCF.

Of the previously known surface molecules, the most strikingly up regulated was CESP. Supporting the original report that this is an epimastigote specific protein [26], our iTRAQ data shows that CESP was up regulated more than 11 fold after differentiation to EMF from PCF (Table 3). It has been hypothesized that CESP may be involved in adherence of EMF to the mouthparts of tsetse [26]. The high levels of CESP observed in EMF and its detection on the cell surface lend support to this idea and suggest that CESP structure-function relationships be examined as a priority in tsetse–trypanosome interactions.

The iTRAQ data indicated that another well known surface molecule of insect forms of *T. congolense*, GARP [17,21] was up-regulated more than two fold in EMF although this was only seen in one replicate. Since by immunoblotting (Fig. 4A) GARP was clearly detectable in both PCF and EMF (and most highly expressed in EMF), the poor quality of the iTRAQ results imply that the peptides either were not released by trypsin cleavage or did not ionize well. The pattern of GARP expression observed with both methods is consistent with data reported previously [32] showing that GARP is expressed early after differentiation to PCF from BSF after which it decreases on established PCF, then is expressed to even higher levels in EMF.

In *T. brucei*, Jackson et al. [33] used cDNA expression to identify the EP procyclin and another protein they called procyclic stage surface antigen, now known as “procyclic form surface glycoprotein”. This new and previously unidentified stage-specific surface antigen had the features of a typical transmembrane glycoprotein but with an unusual cytoplasmic extension composed of a proline-rich tandem repeat. Our iTRAQ analysis revealed that a similar molecule was expressed in *T. congolense* (TcIL3000.10.9440) and that this molecule was up regulated in EMF (2.23 fold increase in PCF from BSF and 4.61 fold increase in EMF from PCF) (Table 3). The *T. congolense* molecule found in our iTRAQ analysis shows 61% sequence identity and 72% similarity to the *T. brucei* molecule. This protein is very unusual. Throughout the sequence there are numerous instances of the same amino acid repeated two (VV, FF, CC, GG, SS, DD, RR, TT, EE, QQ, LL, AA) or three (VVV, RRR, SSS, GGG) times in a row. As seen in the *T. brucei* protein, the final fifth of this protein is proline rich and has several, di-, tri- and tetra-proline repeats.

Another trypanosome surface protein previously reported in both *T. congolense* [34] and *T. brucei* [35] is the major surface metalloprotease (MSP), also called GP63. MSP has been more extensively studied in *T. brucei*, which has a minimum of three classes of MSP genes, *TbMSP*-A, -B and -C, based on sequence differences and differential expression during the *T. brucei* life cycle [36]. All three classes have been shown by northern blot analysis to be transcribed in BSF, whereas only MSP-B RNA was detected in PCF. TbMSP-B protein is absent in BSF, but occurs early in BSF → PCF differentiation and is known to function in removal of VSG during this transition [37]. The functions of TbMSP-A and C are unknown. The *T. congolense* genome has at least five MSP gene classes (*TcoMSP*-A to -E), the first three of which correspond to the three known classes in *T. brucei* [34]. The sequence differences in the MSP classes of both organisms suggest that each class has distinct substrates and biological functions. Our iTRAQ data identified a *T. congolense* MSP that falls into the *TcoMSP*-B1 group and was, surprisingly, most highly expressed in EMF where it was 5.0 fold higher than in PCF. This is the first demonstration of a MSP in the EMF of either trypanosome species and represents an interesting area for further investigation.

Two surface transport molecules showed a striking expression profile throughout the *T. congolense* life cycle. A putative amino acid transporter (TcIL3000.10.13970) and a pteridine transporter (TcIL3000.0.56730) were both drastically up-regulated in EMF (17 and 11 fold respectively) followed by a sharp decrease upon transition to MCF (20 and 6 fold respectively). This increase in expression of these transporters in the EMF may reflect the parasite's response to the scarce supply of nutrients in the tsetse mouthparts.

Another previously identified surface molecule in *T. congolense* insect forms is the major surface procyclin, a GPI-anchored protein consisting almost entirely of heptapeptide repeats (EPGENGT) that has been reported to be expressed continuously on procyclic forms in the tsetse midgut [38]. At first we were surprised that this unusual protein was not detected in our iTRAQ experiments since it was clearly expressed on the surface of both PCF and EMF as determined by flow cytometry and immunofluorescence microscopy using a monoclonal antibody that recognized the heptapeptide repeat (data not shown). In *T. congolense* IL3000 this protein contains only four potential trypsin cleavage sites so perhaps ineffective trypsin cleavage and a dearth of other released peptides accounts for the lack of MS/MS data. Indeed, trypsin cleavage would only release two peptides with the appropriate masses for MS detection. Both peptides are quite hydrophobic and each has a glutamate that could cancel the charge from the arginine and lysine potentially inhibiting their ionization.

Our iTRAQ data showed a marked up regulation of a haptoglobin–hemoglobin receptor [39] in EMF (11.5 fold increase from procyclic forms – Table 3). This can perhaps be explained by the requirement for this form of the parasite to scavenge heme in the nutrient scarce environment of the tsetse mouthparts. In *T. brucei*, this receptor normally binds the haptoglobin–hemoglobin complex at high affinity, allowing uptake of heme and supporting growth of the parasites. In humans, the trypanosome receptor also binds hemoglobin–haptoglobin-related protein complexes that are part of the high-density lipoprotein (HDL) particles containing trypanolytic apolipoprotein L1 [39]. Thus this haptoglobin–hemoglobin receptor binds HDLs and contributes to killing of susceptible parasites [39]. Its role in *T. congolense* has not been studied but presumably, if expressed in bloodstream forms as expected, it would explain in part why this cattle pathogen does not infect humans.

## 4 Conclusions

The study described followed changes in protein expression through all four major stages of the *T. congolense* life cycle. It is not feasible to isolate procyclic forms, epimastigote forms or metacyclic forms of trypanosomes of any species from infected tsetse flies in numbers sufficient for analysis of their proteins. Thus we used *T. congolense* IL3000, a parasite that allows production of the major life cycle stages *in vitro* in sufficient amounts for protein analysis by mass spectrometry. Using iTRAQ-MS/MS we were able to reliably identify a total of 1291 proteins, representing ∼14% of the predicted *T. congolense* proteome. Our data revealed changes in the expression level of many proteins involved in stage-specific metabolic pathways as expected. Much more interesting was the identification of several previously unknown proteins and the drastic differential expression of several surface proteins and glycoproteins, both of known and unknown function. To our knowledge this study represents the first large scale sequence-driven proteomic analysis of all four major life cycle stages for any trypanosome species. Only a few of the proteins identified by this work have been discussed in this manuscript. As a starting point for future thought, Table 4 contains a list of all proteins (which have not already been mentioned in other tables) that show at least a 10 fold change in expression between any two life cycle stages. Ultimately, any researcher wishing to examine the information for particular proteins will have to look at the supplementary Excel sheets or the raw ProteinPilot files. We focused on those proteins of most interest to us or that showed large expression changes. However, many proteins that exhibit much lower differential expression may be of interest and of some biological significance. The data will provide a valuable resource for exploring trypanosome differentiation and/or host–parasite interactions.

## Figures and Tables

**Table 1:** Expression of variant surface glycoproteins in *T. congolense* IL3000 life cycle stages.

*T. congolense* accession no.	BLAST hit accession no.	BLAST hit protein name	BSF → PCF	PCF → EMF	EMF → MCF	MCF → BSF
TcIL3000.0.17670	emb|CAA55938.1	VSG^a^(mVSG6)	DNR^b^	DNR	8.09 (0.95)^c^	0.19 (0.03)
TcIL3000.0.18160	gb|AAA16436.1	VSG	DNR	1.59 (0.25)	6.50 (2.12)	0.13 (0.03)
TcIL3000.0.24660	emb|CAA55938.1	VSG(mVSG3)	0.87 (0.02)	1.76 (0.67)	6.52 (2.37)	0.10 (0.04)
TcIL3000.0.25970	gb|AAA16962.1	VSG	0.12 (0.11)	DNR	8.61 (6.36)	DNR
TcIL3000.0.28420	Gb|AAA16959.1	VSG(IL3000)	0.16 (0.06)	1.45 (0.50)	0.72 (0.25)	8.01 (3.20)
TcIL3000.0.41540	emb|CAA55938.1	VSG	0.10 (0.03)	1.75 (0.20)	1.66 (0.17)	4.61 (1.33)
TcIL3000.0.12850^d^	emb|CAA55938.1	VSG(mVSG10)	DNR	1.71	5.98	0.22
TcIL3000.0.40130	emb|CAJ16171.1	VSG	1.50	0.20	5.50	0.60
TcIL3000.0.58720	gb|AAA16961.1	VSG	0.60	DNR	6.00	DNR
TcIL3000.0.58930	emb|CAQ57406.1	VSG	0.80	0.50	2.50	0.85
TcIL3000.0.58960	emb|CAA55938.1	VSG	0.15	DNR	3.70	DNR

a VSG = variant surface glycoprotein. The name of the VSG is indicated in parenthesis if it has been detected previously [10].

b DNR = data not reliable.

c Standard deviation shown in brackets.

d The VSGs in the shaded areas were detected in two or three biological replicates but the differential expression data were reliable only for one of the replicates.

**Table 2:** Discovery of new proteins in *T. congolense* IL3000 life cycle stages.

*T. congolense* accession no.	BLAST hit accession no.	BSF → PCF	PCF → EMF	EMF → MCF	MCF → BSF
TcIL3000.0.22490	No hits^a^	DNR^b^	DNR	DNR	DNR
TcIL3000.0.28430	No significant hits	0.07 (0.06)^c^	DNR	DNR	17.91 (17.63)
TcIL3000.0.38630	No significant hits	0.15 (0.07)	DNR	8.47 (3.34)	0.62 (0.03)
TcIL3000.0.51090	No significant hits	5.05 (3.63)	0.19 (0.10)	0.17 (<0.00)	5.75 (3.81)
TcIL3000.2.410	No significant hits	DNR	DNR	DNR	DNR
TcIL3000.7.3440	No significant hits	DNR	5.44 (3.72)	0.18 (0.04)	DNR

a No sequence similarity with any protein sequence in the non-redundant database.

b DNR = data not reliable.

c Standard deviation shown in brackets.

**Table 3:** Expression of select surface membrane proteins in *T. congolense* IL3000 life cycle stages.

*T. congolense* accession no.	BLAST hit accession no.	BLAST hit protein name	BSF → PCF	PCF → EMF	EMF → MCF	MCF → BSF
TcIL3000.0.06380	Tb927.8.1610	GP63^a^	DNR^b^	5.03 (3.48)^c^	0.20 (0.19)	DNR
TcIL3000.0.15620	dbj|BAF91109.1	CESP^d^	DNR	11.11 (8.85)	0.17 (0.13)	DNR
TcIL3000.0.22060	Tb927.2.3300	ISG65^e^	0.32 (0.16)	DNR	8.48 (3.78)	0.29 (0.02)
TcIL3000.0.45520	gb|ABB22183.1	ISG75	DNR	DNR	4.73 (2.57)	0.64 (0.11)
TcIL3000.0.56730	Tb927.1.2880	Pteridine transporter	DNR	10.51 (6.53)	0.18 (0.14)	DNR
TcIL3000.10.9440	gb|AAA30244.11	Surface glycoprotein^f^	2.23 (0.78)	4.61 (2.19)	0.59 (0.09)	0.30 (0.02)
TcIL3000.10.2930	Tb927.6.440	Hp–Hb receptor^g^	DNR	11.46 (8.78)	0.17 (0.11)	0.86 (0.06)
TcIL3000.10.13970	Tb927.4.3990	Amino acid transporter	DNR	17.43 (5.02)	0.05 (0.03)	DNR

a Major surface metalloprotease GP63 [34,35].

b DNR = data not reliable.

c Standard deviation shown in brackets.

d CESP = congolense epimastigote specific protein [26].

e ISG = invariant surface glycoprotein [30,31].

f Procylic form surface glycoprotein [33].

g Hp–Hb = haptoglobin–hemoglobin [39].

**Table 4:** Proteins showing an average of at least a 10 fold change in expression between any two life cycle stages.

*T. congolense* accession no.	BLAST hit accession no.	BLAST hit protein name	BSF → PCF	PCF → EMF	EMF → MCF	MCF → BSF
TcIL3000.0.02370	Tb927.7.440	Hypothetical protein	DNR^a^	13.11 (2.37)^b^	0.07 (0.02)	DNR
TcIL3000.0.50300	Tc00.1047053511253.31	Kinesin-like protein	DNR	18.53 (14.79)	0.15 (0.15)	DNR
TcIL3000.0.50590	Tb11.03.0230	Isocitrate dehydrogenase	DNR	13.61 (11.34)	0.49 (0.17)	0.19 (0.01)
TcIL3000.10.10170	Tb10.389.1810	Kynurenine aminotransferase	10.81 (6.05)	0.16 (0.11)	DNR	DNR
TcIL3000.11.8970	Tb11.01.0110	Hypothetical protein	DNR	12.62 (8.99)	0.14 (0.07)	DNR
TcIL3000.7.5580	Tb927.7.6920	Hypothetical protein	DNR	12.58 (4.37)	0.11 (0.05)	DNR

a DNR = data not reliable.

b Standard deviation shown in brackets.

## Appendix A Supplementary data

**Appendix A**. Excel sheet 1 contains all proteins identified by iTRAQ and sorted by replicate; Excel sheet 2 shows only those proteins present in 2 or 3 replicates, sorted by accession number. Expression data have been averaged; Excel sheet 3 contains the same data as Excel sheet 2 although the proteins are organized by their putative function. All proteins that showed positive or negative changes in expression (with high confidence, regardless of intensity) are highlighted in red and blue, respectively. All uncertain expression data have been designated “data not reliable (DNR)”.

**Appendix B, C, D**. ProteinPilot files with the raw iTRAQ analysis of replicate 1, 2, and 3, respectively.
